# Evaluating the impact of restored nonverbal communication on medical service quality and patient loyalty in the post-pandemic era

**DOI:** 10.3389/fpubh.2026.1817920

**Published:** 2026-07-24

**Authors:** Jebum Kim, Lina Wen, Sangyoon Lim

**Affiliations:** 1 Department of Global Culture and Management, Calvin University, Yongin-si, Republic of Korea; 2 School of Economics and Management, Qingdao Agricultural University, Qingdao, China; 3 Seoul National University Bundang Hospital, Seongnam-si, Republic of Korea

**Keywords:** evaluation, flow, loyalty, medical service quality, nonverbal communication, patient experience

## Abstract

**Introduction:**

The transition to the endemic era has restored nonverbal communication in healthcare settings. This study investigates healthcare consumers’ perceptions of Medical Service Quality (MSQ) in this context, specifically examining the impact of restored nonverbal cues on flow, satisfaction, and loyalty.

**Methods:**

A cross-sectional online survey was conducted with 408 healthcare consumers in South Korea who visited medical institutions after the lifting of mask mandates. Structural Equation Modeling (SEM) was employed to test the hypothesized relationships between MSQ dimensions, Technology Acceptance Model (TAM) constructs, and flow.

**Results:**

The analysis revealed that Responsiveness (*β* = 0.547, *p* < 0.001) and Accessibility (*β* = 0.286, *p* = 0.028) significantly enhanced Perceived Usefulness, while Responsiveness (*β* = 0.442, *p* < 0.001), Tangibles (*β* = 0.358, *p* < 0.001), and Accessibility (*β* = 0.344, *p* < 0.001) positively influenced Perceived Ease of Use. Notably, Flow was identified as a key mediator linking service quality to Satisfaction (*β* = 0.142, *p* = 0.024) and Loyalty (*β* = 0.261, *p* < 0.001).

**Conclusion:**

Restored nonverbal communication is not merely a return to the past but a qualitative enhancement of the service environment that drives patient loyalty. Healthcare providers should shift from functional service delivery to patient experience strategies that leverage nonverbal competence as a core component of quality improvement.

## 1 Introduction

The transition from the COVID-19 pandemic to the endemic phase has brought a pivotal change to the healthcare environment: the lifting of mandatory mask-wearing policies. For over three years, face masks served as a critical barrier against infection but simultaneously acted as a barrier to communication (1). During the pandemic, the occlusion of facial expressions significantly limited nonverbal cues, such as smiles, grimaces, and emotional nuances, which are essential for building rapport and trust between patients and medical staff (1). Consequently, healthcare consumers experienced a period of “communication deficit,” where interactions were strictly functional and devoid of emotional depth (2).

Now, with the restoration of face-to-face interactions without masks, healthcare consumers are re-experiencing the full spectrum of nonverbal communication. While extensive literature has documented the negative impact of masks on communication during the pandemic (3, 4), there is a paucity of research examining the qualitative restoration of nonverbal communication in the post-pandemic era. Specifically, it remains unclear how the rediscovery of these nonverbal cues influences patients’ evaluation of medical service quality (MSQ) and their psychological engagement. In the context of healthcare, where service quality is often judged by the empathy and responsiveness of providers (5), the return of nonverbal communication is not merely a procedural change but a qualitative enhancement of the service environment (6).

To address this gap, this study applies the Technology Acceptance Model (TAM) and Flow theory to a non-technical service context. While TAM is traditionally used for information systems (7), its application here is justified by viewing the “restored medical service encounter” as a new service system that patients must cognitively re-evaluate and “accept.” In this framework, Perceived Usefulness and Ease of Use represent the patient’s cognitive assessment of whether unmasked communication effectively facilitates their treatment process. Furthermore, effective nonverbal engagement is posited as a catalyst for “Flow” a state of optimal immersion where the patient feels a deep emotional connection with the provider.

By focusing on this specific transition period, this study provides timely and practical implications for managing patient experience (PX) strategies. Furthermore, it offers a distinct perspective for Quality Improvement (QI) leaders to redesign service protocols that integrate nonverbal competence not just as a soft skill, but as a standard for patient safety and satisfaction.

## 2 Methodology

### 2.1 Theoretical framework and hypotheses

Medical Service Quality (MSQ) is conceptualized as the patient’s comprehensive evaluation of the healthcare delivery process, representing the extent to which a provider meets the patient’s expectations through professional clinical encounters (8). Unlike general services, where quality is often determined at the point of delivery, MSQ is a longitudinal assessment influenced by treatment outcomes and the specific behaviors of healthcare providers over time (9). While the SERVQUAL model, comprising tangibles, reliability, responsiveness, empathy, and assurance, is the representative framework for measurement (10), definitions of MSQ vary across clinical contexts, ranging from broad health promotion to narrow medical consultations (8). Previous research has identified diverse critical factors, including safety and Tangibles (11), Responsiveness and assurance (8), and expertise and accessibility (9, 12). Based on these precedents, this study adapts four dimensions relevant to the post-pandemic interaction: Expertise, Accessibility, Responsiveness, and Tangibles.

The Technology Acceptance Model (TAM), established by Davis (13), introduces Perceived Usefulness (PU) and Perceived Ease of Use (PEOU) as core constructs. PU is defined as the degree to which an individual believes that using a specific system or service will enhance their performance and efficiency, whereas PEOU represents the level of effortlessness associated with that interaction. Although initially applied to information technology, TAM’s scope has expanded significantly into healthcare domains, including e-health and clinical management systems (14). Research among healthcare professionals and patients has consistently demonstrated that MSQ dimensions significantly influence these cognitive appraisals (15, 16).

Furthermore, Flow theory describes a psychological state of deep immersion and intrinsic enjoyment where an individual becomes fully absorbed in an activity (17). It is characterized as a multifaceted concept involving curiosity and challenge, leading to persistent engagement even in the absence of external rewards (18). In the healthcare context, Flow acts as a significant mediator; patients who experience immersion during consultations are more likely to report higher levels of Satisfaction and Loyalty (19, 20). Satisfaction occurs when service expectations are met, leading to positive emotional responses regarding clinical quality and revisiting intentions (21, 22). Loyalty extends this relationship through emotional bonds and trust, which are essential for long-term institutional retention and profitability (23, 24).

Based on this integrated framework, we justify the application of TAM and Flow theory to the “restored” unmasked medical encounter. The following hypotheses are proposed:

*H1*: MSQ positively influences Perceived Usefulness.

*H2*: MSQ positively influences Perceived Ease of Use.

*H3*: Perceived Usefulness has a significant positive effect on Flow.

*H4*: Perceived Ease of Use has a significant positive effect on Flow.

*H5*: Flow positively affects Satisfaction.

*H6*: Flow positively affects Loyalty.

*H7*: Satisfaction positively affects Loyalty.

**Figure 1:**
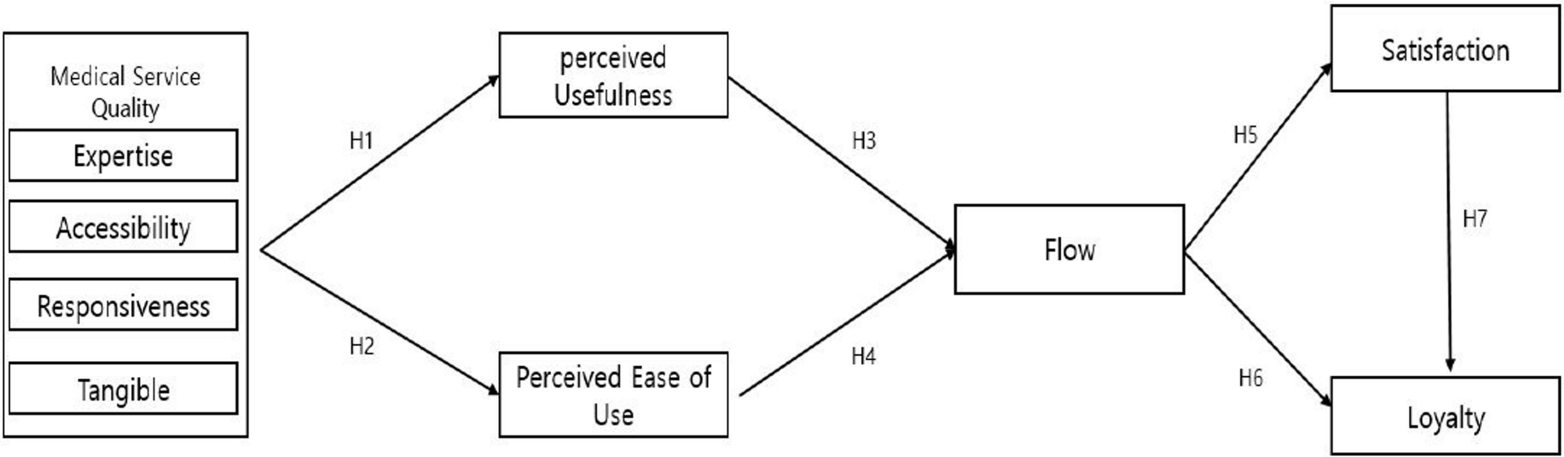
Research model.

The structural relationships between these hypothesized variables are illustrated in the proposed research model (Figure 1).

### 2.2 Research design and data collection

A cross-sectional online survey was conducted from November 20 to December 18, 2024, targeting 408 healthcare consumers in the southern region of Gyeonggi Province, South Korea. To ensure the validity of the post-pandemic context, participation was limited to individuals who visited a medical institution after May 11, 2023 the date mandatory mask policies were lifted in South Korea. A 5-point Likert scale (1 = Strongly Disagree, 5 = Strongly Agree) was utilized to balance measurement precision with respondent fatigue, consistent with established behavioral healthcare research.

### 2.3 Measurement instruments and validation

The questionnaire consisted of 36 items adapted from validated scales. MSQ was measured using four dimensions adapted from the SERVQUAL framework (8, 9, 11, 12). TAM constructs were adapted from Davis (13) and Venkatesh and Davis (14). Flow items were drawn from Hoffman and Novak (17) and Lavoie et al. (18), and outcome variables (Satisfaction and Loyalty) were adapted from healthcare literature (21–24). The specific operational definitions and the primary references for each variable are summarized in Table 1.

**Table 1:** Operational definition of variables.

Variable	Operational Definition	References
Medical service quality – Expertise	The degree to which patients perceive the medical staff’s clinical competence and professionalism through nonverbal cues (e.g., confident posture, eye contact).	(8, 9, 11, 12)
Medical service quality – Accessibility	The degree of psychological openness and approachability felt by patients due to the restoration of unmasked face-to-face interactions.	
Medical service quality – Responsiveness	The extent to which medical staff are perceived to provide immediate and empathetic reactions to patient needs through facial expressions and gestures.	
Medical service quality – Tangible	The perception of the physical environment and the neatness of the medical staff’s unmasked appearance as signals of a safe and professional service.	
Perceived usefulness	The degree to which a patient believes that restored nonverbal communication improves the effectiveness and quality of the medical consultation.	(13, 14)
Perceived ease of use	The degree to which a patient feels that the medical interaction is effortless and the communication is clear due to visible facial cues.	
Flow	The state of psychological immersion and deep engagement experienced by the patient during the medical encounter.	(17, 18)
Satisfaction	The overall positive emotional evaluation of the healthcare service experienced in the post-pandemic (endemic) context.	(21–24)
Loyalty	The patient’s behavioral intention to revisit the healthcare institution and recommend it to others based on their experience.	

To address potential Common Method Bias (CMB), Harman’s single-factor test was performed prior to the analysis; the results indicated that no single factor accounted for more than 50% of the variance, confirming CMV was not a significant threat.

### 2.4 Analytical methods

Data were analyzed using SPSS 29.0 and AMOS 29.0. The analysis included descriptive statistics, Confirmatory Factor Analysis (CFA) to assess reliability and validity, and Structural Equation Modeling (SEM) to test the hypothesized structural relationships.

## 3 Results

### 3.1 Demographic characteristics

The demographic profile of the 408 respondents is summarized in Table 2. The gender distribution was relatively balanced, with 55.1% male (*n* = 225) and 44.9% female (*n* = 183) respondents. Regarding age, the largest group was individuals in their 30s (35.5%), followed by those in their 20s (26.2%) and 40s (23.0%). In terms of occupation, company employees constituted the majority (29.2%), followed by service employees (19.4%). The most common monthly income bracket was KRW 3.0–4.0 million (25.5%).

**Table 2:** Demographic characteristics.

Characteristic	Category	Frequency (*n*)	Percentage (%)
Gender	Male	225	55.1
	Female	183	44.9
Age	20s	107	26.2
	30s	145	35.5
	40s	94	23.0
	50s	53	13.0
	60 and above	9	2.2
Occupation	Company employee	119	29.2
	Service employee	79	19.4
	Technical employee	42	10.3
	Professional	53	13.0
	Student	29	7.1
	Other	86	21.1
Monthly income (KRW)	Less than 2,000,000	61	15.0
	2,000,000–2,999,999	76	18.6
	3,000,000–3,999,999	104	25.5
	4,000,000–4,999,999	100	24.5
	5,000,000 and above	67	16.4
Total		408	100

### 3.2 Measurement model assessment

To evaluate the reliability and validity of the measurement model, a Confirmatory Factor Analysis (CFA) was conducted. As shown in Table 3, the model fit indices indicated a satisfactory fit: χ2/df = 1.690, GFI = 0.882, TLI = 0.967, CFI = 0.971, SRMR = 0.028, and RMSEA = 0.041.

**Table 3:** Confirmatory factor analysis.

Construct	Factor loadings	Cronbach’s α	AVE	CR
Expertise	0.928	0.912	0.84	0.96
	0.925			
	0.908			
	0.909			
Accessibility	0.916	0.890	0.81	0.94
	0.903			
	0.911			
	0.861			
Responsiveness	0.909	0.896	0.82	0.95
	0.92			
	0.893			
	0.901			
Tangible	0.887	0.886	0.78	0.93
	0.874			
	0.893			
	0.875			
Perceived usefulness	0.927	0.915	0.85	0.96
	0.927			
	0.917			
	0.917			
Perceived ease of use	0.924	0.919	0.84	0.96
	0.938			
	0.911			
	0.897			
Flow	0.896	0.892	0.80	0.94
	0.903			
	0.892			
	0.884			
Satisfaction	0.912	0.906	0.83	0.95
	0.919			
	0.912			
	0.893			
Loyalty	0.903	0.878	0.81	0.95
	0.911			
	0.917			
	0.874			

χ^2^/df = 1.690, GFI = 0.882, TLI = 0.967, CFI = 0.971, SRMR = 0.028, RMSEA = 0.041.

Internal consistency was assessed using Cronbach’s *α* All constructs exhibited α values ranging from 0.87 to 0.92, exceeding the recommended threshold of 0.70. Convergent validity was confirmed as the Average Variance Extracted (AVE) for all constructs exceeded 0.50, and Composite Reliability (CR) values were greater than 0.70.

Discriminant validity was assessed by comparing the square root of the AVE with the correlations between constructs. As presented in Table 4, the square roots of the AVEs (diagonal elements) were greater than the inter-construct correlations, supporting discriminant validity.

**Table 4:** Discriminant validity analysis results.

Construct	Expertise	Accessibility	Responsiveness	Tangible	Perceived Usefulness	Perceived Ease of Use	Flow	Satisfaction	Loyalty
Expertise	(0.917)								
Accessibility	0.774**	(0.900)							
Responsiveness	0.816**	0.743**	(0.906)						
Tangible	0.755**	0.800**	0.761**	(0.883)					
Perceived usefulness	0.778**	0.731**	0.786**	0.723**	(0.922)				
Perceived ease of use	0.754**	0.786**	0.774**	0.791**	0.789**	(0.917)			
Flow	0.742**	0.697**	0.758**	0.699**	0.732**	0.702**	(0.894)		
Satisfaction	0.756**	0.748**	0.793**	0.764**	0.784**	0.789**	0.749**	(0.911)	
Loyalty	0.729**	0.724**	0.742**	0.734**	0.736**	0.772**	0.696**	0.725**	(0.900)

*p < 0.05, **p < 0.01, ***p < 0.001.

### 3.3 Structural model assessment and hypothesis testing

The structural model was examined to test the proposed hypotheses (H1–H7). The results of the path analysis are summarized in Table 5.

**Table 5:** Hypothesis testing results.

Path	Estimate	S. E.	C. R.	*p*	Decision
Expertise → Perceived usefulness	0.194	0.098	1.982	0.047	Supported
Accessibility → Perceived usefulness	0.286	0.13	2.2	0.028	Supported
Responsiveness → Perceived usefulness	0.547	0.104	5.278	***	Supported
Tangible → Perceived usefulness	0.031	0.1	0.316	0.752	Not supported
Expertise → Perceived ease of use	−0.059	0.106	−0.562	0.574	Not supported
Accessibility → Perceived ease of use	0.344	0.101	3.416	***	Supported
Responsiveness → Perceived ease of use	0.442	0.107	4.115	***	Supported
Tangible → Perceived ease of use	0.358	0.107	3.361	***	Supported
Perceived usefulness → Flow	0.373	0.068	5.511	***	Supported
Perceived ease of use → Flow	0.430	0.054	7.993	***	Supported
Flow → Satisfaction	0.142	0.063	2.257	0.024	Supported
Flow → Loyalty	0.261	0.069	3.799	***	Supported
Satisfaction → Loyalty	0.540	0.069	7.811	***	Supported

*p < 0.05, **p < 0.01, ***p < 0.001.

Regarding Perceived Usefulness (H1), the dimensions of Responsiveness (*β* = 0.547, *p* < 0.001), Accessibility (*β* = 0.286, *p* = 0.028), and Expertise (*β* = 0.194, *p* = 0.047) showed significant positive effects. However, Tangibles (*β* = 0.031, *p* = 0.752) did not have a significant effect. Regarding Perceived Ease of Use (H2), Responsiveness (*β* = 0.442, *p* < 0.001), Tangibles (*β* = 0.358, *p* < 0.001), and Accessibility (*β* = 0.344, *p* < 0.001) showed significant positive effects. Conversely, Expertise (*β* = −0.059, *p* = 0.574) did not exert a significant influence.

For the TAM, Flow, and Outcome relationships (H3–H7), both Perceived Ease of Use (*β* = 0.430, *p* < 0.001) and Perceived Usefulness (*β* = 0.373, *p* < 0.001) significantly increased Flow, supporting H3 and H4. Subsequently, Flow had a significant positive impact on Loyalty (*β* = 0.261, *p* < 0.001) and Satisfaction (*β* = 0.142, *p* = 0.024), supporting H5 and H6. Finally, Satisfaction was found to be a strong predictor of Loyalty (*β* = 0.540, *p* < 0.001), supporting H7.

## 4 Discussion and conclusion

### 4.1 Interpretation of key findings

The empirical results of this study provide a nuanced understanding of how healthcare consumers perceive Medical Service Quality (MSQ) in the context of restored nonverbal communication. First, regarding Perceived Usefulness, Expertise, Accessibility, and Responsiveness were found to have significant positive effects, whereas Tangibles did not exhibit a significant influence. This suggests that while consumers value the clinical and communicative competence of providers, they do not necessarily perceive the physical environment as a primary driver of the “utility” or effectiveness of the unmasked consultation process. Conversely, for Perceived Ease of Use, Accessibility, Responsiveness, and Tangibles were significant drivers, while Expertise was not. This indicates that high-level clinical expertise, while useful, is often perceived as complex and may not inherently contribute to the “ease” of the interaction unless accompanied by approachable nonverbal cues.

Furthermore, our analysis confirmed that both Perceived Usefulness and Perceived Ease of Use significantly enhance Flow, aligning with prior research that identifies these cognitive appraisals as precursors to psychological immersion (25, 26). This immersion, or “Flow,” was shown to be a critical driver of Satisfaction and Loyalty, echoing findings in specialized healthcare sectors such as beauty medical services (27). Finally, the significant positive relationship between Satisfaction and Loyalty reinforces the established paradigm in both general and university hospital settings (27, 28), suggesting that emotional fulfillment remains the strongest predictor of long-term patient retention in the endemic era.

### 4.2 Theoretical and practical implications

Theoretically, this research contributes to the expansion of MSQ literature by focusing on the transition from the pandemic to the endemic phase. While previous studies extensively analyzed the constraints of the pandemic era (29, 30), there has been a paucity of research on the restoration of nonverbal cues. By empirically validating the role of nonverbal restoration, this study bridges a critical gap in contemporary healthcare management.

Practically, our findings offer several strategic insights. First, despite the non-significant path from Tangibles to Usefulness in this study, existing literature emphasizes Tangibles as a crucial determinant of overall satisfaction (31). Therefore, healthcare institutions should not neglect physical service quality even as they prioritize nonverbal training. Second, the lack of a significant link between Expertise and Ease of Use underscores the need for providers to “translate” their technical competence into patient-friendly interactions. This aligns with the necessity of maintaining high clinical standards (32, 33) while ensuring they are communicated in an accessible manner. Lastly, the vital role of Flow in driving behavioral outcomes suggests that hospital administrators should design consultation environments that foster deep patient engagement and emotional rapport.

### 4.3 Limitations and future research

Despite its contributions, several limitations of this study should be addressed in future research:

1. Institutional diversity: While this study targeted general healthcare consumers, it did not differentiate between healthcare institutional types. Future research should conduct comparative analyses between primary, secondary, and tertiary medical institutions to obtain more granular insights into how nonverbal communication varies by clinical setting.
2. Stakeholder roles: This study did not distinguish between different types of consumers, such as patients and their family members. Subsequent studies should categorize respondents by their specific role to understand the nuanced psychological responses of various stakeholders.
3. Clinical specialization: The current data do not account for differences across clinical departments (e.g., orthopedics, internal medicine, or rehabilitation). Future investigations should segment data by medical specialty to identify department-specific service quality drivers.
4. Purpose of treatment: Finally, this study did not distinguish between treatment purposes, such as cosmetic improvement, general health promotion, or surgical intervention. Future research should explore how service quality perceptions shift according to the patient’s underlying motivation for seeking care.

## Data Availability

The original contributions presented in the study are included in the article/supplementary material, further inquiries can be directed to the corresponding author.

## References

[1] Hall JA Horgan TG Murphy NA. Nonverbal communication. Annu Rev Psychol. (2019) 70:271–94. doi: 10.1146/annurev-psych-010418-103145 30256720

[2] Saunders GH Jackson IR Visram AS. Impacts of face coverings on communication: an indirect impact of COVID-19. Int J Audiol. (2021) 60:495–506. doi: 10.1080/14992027.2020.1851401, 33246380

[3] Mheidly N Fares MY Zalzale H Fares J. Effect of face masks on interpersonal communication during the COVID-19 pandemic. Front Public Health. (2020) 8:582191. doi: 10.3389/fpubh.2020.582191, 33363081 PMC7755855

[4] Marler H Ditton A. “I'm smiling back at you”: exploring the impact of mask wearing on communication in healthcare. Int J Lang Commun Disord. (2021) 56:205–14. doi: 10.1111/1460-6984.12578, 33038046 PMC7675237

[5] Lee YW Choi HG Kwon HM. Impact of medical service quality in nursing hospitals on consumption value, attitude, and intention to continue using. Corp Manag Rev. (2020) 11:127–50. doi: 10.20434/KRICM.2020.08.11.3.127

[6] Del Giacco L Anguera MT Salcuni S. The action of verbal and non-verbal communication in the therapeutic alliance construction: a mixed methods approach to assess the initial interactions with depressed patients. Front Psychol. (2020) 11:234. doi: 10.3389/fpsyg.2020.00234, 32153459 PMC7047748

[7] Franque FB Oliveira T Tam C Santini FD. A meta-analysis of the quantitative studies in continuance intention to use an information system. Internet Res. (2021) 31:123–58. doi: 10.1108/INTR-03-2019-0103, 35579975

[8] Akdere M Top M Tekingündüz S. Examining patient perceptions of service quality in Turkish hospitals: the SERVPERF model. Total Qual Manag Bus Excell. (2020) 31:342–52. doi: 10.1080/14783363.2018.1427501

[9] Endeshaw B. Healthcare service quality-measurement models: a review. J Health Res. (2021) 35:106–17. doi: 10.1108/JHR-07-2019-0152, 35579975

[10] Parasuraman A Zeithaml VA Berry LL. A conceptual model of service quality and its implications for future research. J Mark. (1985) 49:41–50. doi: 10.1177/002224298504900403

[11] Lee D. HEALTHQUAL: a multi-item scale for assessing healthcare service quality. Serv Bus. (2017) 11:491–516. doi: 10.1007/s11628-016-0317-2

[12] Le KH La TXP Tykkyläinen M. Service quality and accessibility of healthcare facilities: digital healthcare potential in Ho Chi Minh City. BMC Health Serv Res. (2022) 22:1374. doi: 10.1186/s12913-022-08758-w, 36403031 PMC9675284

[13] Davis FD. "Technology acceptance model: TAM". In: Al-Suqri MN Al-Aufi AS, editors. Information Seeking Behavior and Technology Adoption. Hershey, PA, USA: IGI Global (1989). p. 205–19.

[14] Venkatesh V Davis FD. A theoretical extension of the technology acceptance model: four longitudinal field studies. Manag Sci. (2000) 46:186–204. doi: 10.1287/mnsc.46.2.186.11926

[15] Hogan J Grant G Kelly F O'Hare J. Factors influencing acceptance of robotics in hospital pharmacy: a longitudinal study using the extended technology acceptance model. Int J Pharm Pract. (2020) 28:483–90. doi: 10.1111/ijpp.12637, 32430998

[16] Bernarto I. The effect of performance expectancy, perceived need, perceived value and perceived ease of use on patient satisfaction and continuance intention in the Halodoc telemedicine service application in Jabodetabek in 2023. JMBI UNSRAT. (2024) 11:460–71. doi: 10.35794/jmbi.v11i1.54142

[17] Hoffman DL Novak TP. Flow online: lessons learned and future prospects. J Interact Mark. (2009) 23:23–34. doi: 10.1016/j.intmar.2008.10.003

[18] Lavoie R Main K Stuart-Edwards A. Flow theory: advancing the two-dimensional conceptualization. Motiv Emot. (2022) 46:38–58. doi: 10.1007/s11031-021-09911-4

[19] Aboelmaged MG. Predicting the success of twitter in healthcare: a synthesis of perceived quality, usefulness and flow experience by healthcare professionals. Online Inf Rev. (2018) 42:898–922. doi: 10.1108/OIR-01-2017-0018

[20] Gómez-Rico M Santos-Vijande ML Molina-Collado A Bilgihan A. Unlocking the flow experience in apps: fostering long-term adoption for sustainable healthcare systems. Psychol Mark. (2023) 40:1556–78. doi: 10.1002/mar.21824

[21] Liu S Li G Liu N Wang H. The impact of patient satisfaction on patient loyalty with the mediating effect of patient trust. Inquiry (United States). (2021) 58:00469580211007221. doi: 10.1177/00469580211007221 PMC8040618 33834860

[22] Radu F Radu V Turkeș MC Ivan OR Tăbîrcă AI. A research of service quality perceptions and patient satisfaction: case study of public hospitals in Romania. Int J Health Plann Manag. (2022) 37:1018–48. doi: 10.1002/hpm.3375, 34787918

[23] Nguyen TLH Nagase K. Patient satisfaction and loyalty to the healthcare organization. Int J Pharm Healthc Mark. (2021) 15:496–515. doi: 10.1108/IJPHM-02-2020-0011

[24] AlOmari F Hamid AB. Strategies to improve patient loyalty and medication adherence in Syrian healthcare setting: the mediating role of patient satisfaction. PLoS One. (2022) 17:e0272057. doi: 10.1371/journal.pone.0272057 36399483 PMC9674161

[25] Wang S Sun W Liu J Nah K Yan W Tan S. The influence of AR on purchase intentions of cultural heritage products: the TAM and flow-based study. Appl Sci. (2024) 14:7169. doi: 10.3390/app14167169

[26] Huang L Liu M. Predicting flow state and learning engagement in GAI-powered virtual influencer-mediated informal foreign language learning. Eur J Educ. (2026) 61:e70575. doi: 10.1111/ejed.70575

[27] Kwon M Sung H. Effects of e-service quality in beauty medical services on flow, satisfaction and loyalty. Prod Stud Int J. (2023) 37:29–60.

[28] Heo E Oh J Jang H. The structural relationship between trust, customer satisfaction, and loyalty of the medical service quality and empathy ability. Korean Bus Educ Rev. (2017) 32:1–22. doi: 10.23839/kabe.2017.32.6.1

[29] Prante FJ Bramucci A Truger A. Decades of tight fiscal policy have left the health care system in Italy ill-prepared to fight the COVID-19 outbreak. Intereconomics. (2020) 55:147–52. doi: 10.1007/s10272-020-0886-0, 32536710 PMC7276098

[30] Tuczyńska M Staszewski R Matthews-Kozanecka M Żok A Baum E. Quality of the healthcare services during COVID-19 pandemic in selected European countries. Front Public Health. (2022) 10:870314. doi: 10.3389/fpubh.2022.870314, 35646786 PMC9133554

[31] Turan A Bozaykut-Bük T. Analyzing perceived healthcare service quality on patient related outcomes. Int J Qual Serv Sci. (2016) 8:478–97. doi: 10.1108/IJQSS-04-2015-0042

[32] Fatima I Humayun A Iqbal U Shafiq M. Dimensions of service quality in healthcare: a systematic review of literature. Int J Qual Health Care. (2019) 31:11–29. doi: 10.1093/intqhc/mzy125 29901718

[33] Jonkisz A Karniej P Krasowska D. SERVQUAL method as an “old new” tool for improving the quality of medical services: a literature review. Int J Environ Res Public Health. (2021) 18:10758. doi: 10.3390/ijerph182010758, 34682499 PMC8535625

